# Consultation-oriented teacher support and student innovation literacy: a structural model

**DOI:** 10.3389/fpsyg.2025.1697366

**Published:** 2025-12-09

**Authors:** Wenqian Gao, Baojian Wei, Yan Xiong

**Affiliations:** 1 Wuhan Institute of Shipbuilding Technology, Wuhan, China; 2 School of Nursing, Shandong First Medical University & Shandong Academy of Medical Sciences, Jinan, Shandong, China; 3 College of Education, Capital Normal University, Beijing, China

**Keywords:** educational–psychological consultation, consultation-oriented teacher/counselor support, psychological safety, intrinsic motivation, cognitive load, innovation literacy

## Abstract

Amid the accelerated transformation of higher education, university students face intensifying cognitive demands that challenge both learning and innovation. Drawing upon cognitive load theory and self-determination theory, this study investigates the psychological pathways through which consultation-oriented teacher or counselor support (TS) enhances students’ innovation literacy. A chain-mediation structural equation model (SEM) was developed to examine the sequential relationships among TS, psychological safety (PS), intrinsic motivation (IM), cognitive load (CL), and innovation literacy, while perceived innovation climate was introduced as a contextual moderator. Data were collected from 300 undergraduates at three universities located in Eastern, Central, and Western China using validated questionnaires assessing TS (emotional encouragement, exploratory guidance, feedback support), PS, IM, subjective CL (task complexity and effort), and innovation literacy (cognitive, motivational, and behavioral dimensions). Results indicated that TS significantly improved PS, which in turn enhanced IM and reduced perceived CL. These psychological mechanisms jointly promoted students’ innovation literacy, particularly its cognitive and motivational components, whereas the pathway from IM to observable innovation behavior remained comparatively weak, reflecting a motivational–behavioral gap. Furthermore, perceived innovation climate moderated several key paths, indicating that supportive institutional environments amplify the positive effects of consultation-oriented support on psychological resources and innovative outcomes. Overall, the study establishes an integrated consultation-oriented framework for understanding support-driven innovation in higher education and offers practical implications for consultant training, teacher coaching, and institutional policy aimed at optimizing task design, balancing cognitive demands, and transforming motivation into sustainable innovation behavior.

## 1 Introduction

In the era of AI-driven Knowledge Production 4.0, global data volume continues to expand at an annual rate of 24.4% (IDC, 2024), transforming the foundations of knowledge generation and shifting labor market demands from “knowledge reproducers” to “knowledge creators.” This transformation highlights the need for individuals capable of transcending linear reasoning and cultivating innovative, integrative, and adaptive thinking for the creative transformation of knowledge. Creativity has thus emerged not merely as an artistic trait but as a multidimensional capacity involving originality, problem-solving, and innovation across contexts (Yuan et al., 2022; Torrance, 1966; Cropley and Cropley, 2010). Within higher education, it has become a vital learning outcome for preparing students to thrive in a rapidly evolving digital economy. Employers increasingly expect graduates to demonstrate not only technical expertise but also creative and innovative competencies (Frey and Osborne, 2017). Yet empirical evidence suggests that many graduates still lack creativity and, more critically, awareness of their creative potential (Robinson, 2014; Cao et al., 2024). Recent scholarship further observes that traditional higher education frameworks have struggled to systematically cultivate and assess creative thinking, underscoring the need to embed innovation-oriented learning beyond conventional disciplinary curricula (Karunarathne and Calma, 2024; Kuh, 2009). Research in organizational and educational psychology also reveals that creativity depends not only on individual differences—such as personality traits (Goncalo and Duguid, 2012), cognitive styles (Mathisen et al., 2008), and creative performance (Gong et al., 2013)—but equally on supportive environmental conditions (Hennessey and Amabile, 2010). Collectively, these findings call for a paradigm shift in higher education: from knowledge transmission toward cultivating creative capacity through psychological and pedagogical support mechanisms (Zhang and Tian, 2024).

Despite growing recognition of creativity’s importance, research on creativity education remains fragmented and discipline-specific, often neglecting the pivotal role of teacher support in developing students’ innovative competencies (He et al., 2018; He, 2017). Teachers serve not merely as knowledge providers but as facilitators of students’ motivation, cognition, and emotional safety through value guidance, feedback, and mentorship. Prior studies have established that psychologically safe learning environments foster both engagement and creative performance (Edmondson, 1999; Frazier et al., 2017; Schön, 2017), while intrinsic motivation functions as a key internal driver of creativity (Hennessey and Amabile, 2010; Liu et al., 2016; Amabile, 1983). However, current work on teacher support often remains superficial—focused on classroom atmosphere or satisfaction surveys—without systematically uncovering the psychological and cognitive pathways that link support to creativity. Although the Code of Professional Ethics for University Teachers (Ministry of Education of the People’s Republic of China, 2017) explicitly mandates educators to “stimulate students’ creative spirit and innovative capacity,” empirical understanding of how this mandate translates into practice remains limited. Building on the competency model proposed by Spencer and Spencer (1993), teachers are now expected to expand their professional skillsets to include fostering innovation-friendly environments, employing creative pedagogical methods, and identifying students’ creative potential. To address these gaps, the present study develops and empirically tests an integrated framework linking consultation-oriented teacher support and student innovation literacy. Grounded in cognitive load theory and self-determination theory, the study examines the mediating roles of psychological safety, intrinsic motivation, and cognitive load, as well as the moderating influence of innovation climate. By constructing a multidimensional teacher support model (encompassing learning environment building, creative problem-solving guidance, and potential stimulation) and a student innovation literacy model (covering cognitive, motivational, and behavioral dimensions), this research provides a comprehensive understanding of how teacher support fosters creativity in higher education. Ultimately, the study contributes both theoretical refinement and practical insight—informing teacher professional development, consultation systems, and curriculum innovation aimed at transforming motivation into observable creative behavior (Zhou and George, 2001; Zhou, 2003). Accordingly, the following sections review theoretical and empirical foundations to develop hypotheses.

## 2 Literature review

### 2.1 Theoretical foundations of teacher competency and support

In the mid-twentieth century, psychologist McClelland (1973) introduced the concept of “competency,” arguing that conventional intelligence tests were inadequate for predicting performance in real-world contexts. He proposed that success in professional and daily life depends on a broader integration of knowledge, skills, abilities, and attitudes that enable individuals to achieve specific goals effectively. Building on this foundation, Spencer and Spencer (1993) developed the well-known “iceberg model,” distinguishing explicit competencies—such as knowledge and skills—from implicit ones including values, motivations, and personality traits. This model became a cornerstone in evaluating professional performance and later informed competency-based education and teacher development. Over the past two decades, research on competency models has evolved across four major dimensions: model construction, multidimensional frameworks, assessment methods, and practical applications. For instance, Marrelli et al. (2005) proposed a systematic seven-step model-building process encompassing objective definition, stakeholder engagement, methodological selection, competency identification, model implementation, and ongoing revision. Some scholars expanded this perspective by incorporating four interrelated components—learning, education, society, and technology—into teacher competency models, emphasizing their connection to innovative pedagogy (Zhu et al., 2013; Redeker, 2017). The European Union’s Digital Competence Framework for Educators established a transnational structure that has since been adapted nationally, such as Spain’s Framework for Digital Competence of Educators (European Commission/EACEA/Eurydice, 2019) and Norway’s Digital Competence Framework for Teachers (Norwegian Directorate for Education and Training, 2017), both underscoring the importance of creativity, critical thinking, and problem-solving. Concurrently, studies on teachers’ evolving psychological and behavioral characteristics have contributed to refining these frameworks. Zlatkin-Troitschanskaia et al. (2017) advanced the field by operationalizing competency frameworks into measurable assessment tools combining expert evaluation and student feedback, while Xu et al. (2018) examined how teacher self-efficacy interacts with professional competencies, recommending reflective and targeted training approaches (Shen and Zhang, 2019). These developments have been enriched by motivational and relational perspectives, particularly through Deci and Ryan’s (1985, 2000) self-determination theory, which highlights autonomy, competence, and relatedness as core psychological needs in teaching and learning. Within this framework, Reeve (2006) and Wentzel (1998) demonstrated that autonomy-supportive teaching enhances student engagement and well-being, while Skinner and Belmont (1993) and Pianta (1999) showed that emotionally supportive teacher–student relationships promote psychological safety and academic persistence (Amabile, 2018). Collectively, these findings indicate that teacher competency extends beyond technical expertise to encompass emotional and motivational capacities that foster supportive learning environments and meaningful psychological connection between teachers and students.

### 2.2 Conceptualization of innovation and innovation literacy

The concepts of innovation and creativity have long been central to educational and organizational research, yet their definitions and mechanisms remain complex and contested. Sternberg and Lubart (1999) defined creativity as the capacity to produce ideas or products that are both novel and useful, emphasizing that originality must serve meaningful purposes rather than novelty alone (Sternberg and Lubart, 1999). A consistent theme in the literature is that innovation is not an isolated cognitive act but a dynamic process shaped by social, motivational, and environmental factors. Amabile et al. (1996) demonstrated that creativity flourishes when individuals are intrinsically motivated and work within supportive climates, whereas excessive evaluation or external control inhibits creative expression (Amabile et al., 1996). Complementary findings by Scott and Bruce (1994) revealed that leader support and openness to new ideas predict innovative behavior (Scott and Bruce, 1994), while Rogers (2003) and Walker (2006) emphasized the diffusion and contextual adaptation of innovation within organizational systems, underscoring its collective and systemic nature. Within this broader theoretical evolution, innovation literacy has come to represent an integrated capability encompassing cognitive, social, and technological dimensions that enable learners to transform knowledge into creative and socially valuable outcomes. The Partnership for 21st Century Skills positioned critical thinking and problem-solving at the core of learning and innovation skills, and subsequent frameworks, such as the OECD’s *Future of Education and Skills 2030*, defined innovation as the creation of new value grounded in collaboration, ethics, and sustainability. Empirical developments further linked innovation literacy to technological fluency and adaptive learning, as reflected in PISA 2018 and national curriculum reforms that incorporate analytical reasoning as an indicator of creative readiness. More recent studies have integrated psychological perspectives, highlighting that innovation depends not only on external context but also on internal belief systems. Tierney and Farmer (2002) identified creative self-efficacy as a central determinant of innovative performance, while Runco and Jaeger (2012) clarified that creativity entails both originality and effectiveness, bridging conceptual and practical dimensions. In educational technology contexts, Cropley and Cropley (2010) argued that cultivating innovation literacy requires recognizing creativity within design-based and problem-solving processes rather than isolating it as an abstract skill. Taken together, these strands of research suggest that innovation literacy is best understood as the synthesis of creative cognition, self-efficacy, and technological adaptability, forming an integrated framework that transforms knowledge into meaningful, applicable, and socially impactful innovation across educational and professional settings.

### 2.3 Cultivation and mechanisms of innovation literacy

Research on cultivating innovation literacy has developed along two complementary disciplinary trajectories—psychology and education—each contributing distinct yet interconnected perspectives on how creative potential can be effectively nurtured. From a psychological standpoint, three classic theoretical models have provided enduring guidance. Guilford’s (1967) structure of intellect model identified divergent thinking as the cognitive foundation of creativity, emphasizing flexibility and originality in problem-solving. Torrance (1966) advanced this understanding by designing the Torrance Tests of Creative Thinking, which operationalized creative processes into measurable educational constructs and became a global standard in creativity assessment. Csikszentmihalyi’s (1990) flow theory introduced an experiential dimension, proposing that creativity emerges when challenges align with individual skill levels, producing deep engagement and intrinsic satisfaction. Expanding on these frameworks, Beghetto and Kaufman (2007) proposed the mini-C to Pro-C model, illustrating how everyday creativity can evolve into professional innovation through cross-domain integration and reflective development (Beghetto, 2019). Contemporary research has extended these foundational models into digital and technology-mediated learning environments. Tang et al. (2022) found that interactive online learning enhances creative performance through dynamic teacher–student engagement, while Zhan et al. (2024) confirmed via meta-analysis that problem-solving pedagogies significantly improve creativity, with instructional design serving as a critical mediating factor. Educational research further underscores the social and emotional dimensions of innovation literacy. Zhang (2021) demonstrated that entrepreneurship education and experiential learning activities promote students’ creative competence, and Hou et al. (2023) showed that immersive metaverse environments strengthen innovation literacy through simulation-based learning and experimental interaction. Parallel findings in educational psychology highlight the role of teacher behavior and instructional structure (Hou and Wang, 2023). According to Deci and Ryan (2000), intrinsic motivation thrives when autonomy, competence, and relatedness are fulfilled, and Reeve (2006) emphasized that autonomy-supportive teaching enhances cognitive engagement and self-directed learning. Complementarily, Sweller’s (1988) cognitive load theory, later refined by Sweller et al. (2019), demonstrated that effective instructional design reduces unnecessary cognitive burden and fosters efficient problem-solving. Collectively, these studies suggest that the cultivation of innovation literacy depends on integrating motivational, cognitive, and environmental mechanisms within a coherent learning ecosystem that balances structure with freedom, challenge with support, and individual exploration with collaborative creativity.

### 2.4 The role of teacher support in developing innovation literacy

Building on these insights above, it becomes clear that teacher support operates as a comprehensive mechanism that integrates motivational, cognitive, and emotional processes to foster innovation literacy. By offering structured guidance and psychological safety, teachers enable students to manage cognitive complexity, sustain engagement, and transform intrinsic motivation into creative performance. Consultation-oriented support further provides a balance between cognitive challenge and emotional assurance, allowing learners to explore, take intellectual risks, and apply knowledge in novel ways. Within this perspective, innovation literacy is not simply a reflection of individual ability but a dynamic outcome shaped by the interaction between supportive teaching, intrinsic motivation, and effective cognitive regulation. Grounded in self-determination theory and cognitive load theory, the present study proposes that teacher support enhances innovation literacy through sequential pathways involving psychological safety, intrinsic motivation, and cognitive load reduction. Moreover, it posits that contextual factors such as the perceived innovation climate may strengthen or weaken these effects, highlighting the importance of situational dynamics in shaping innovative outcomes. These theoretical propositions form the foundation for the hypotheses tested in this study, aiming to clarify how motivational and cognitive mechanisms jointly mediate the impact of teacher support on students’ innovation literacy in higher education.

**Figure 1:**
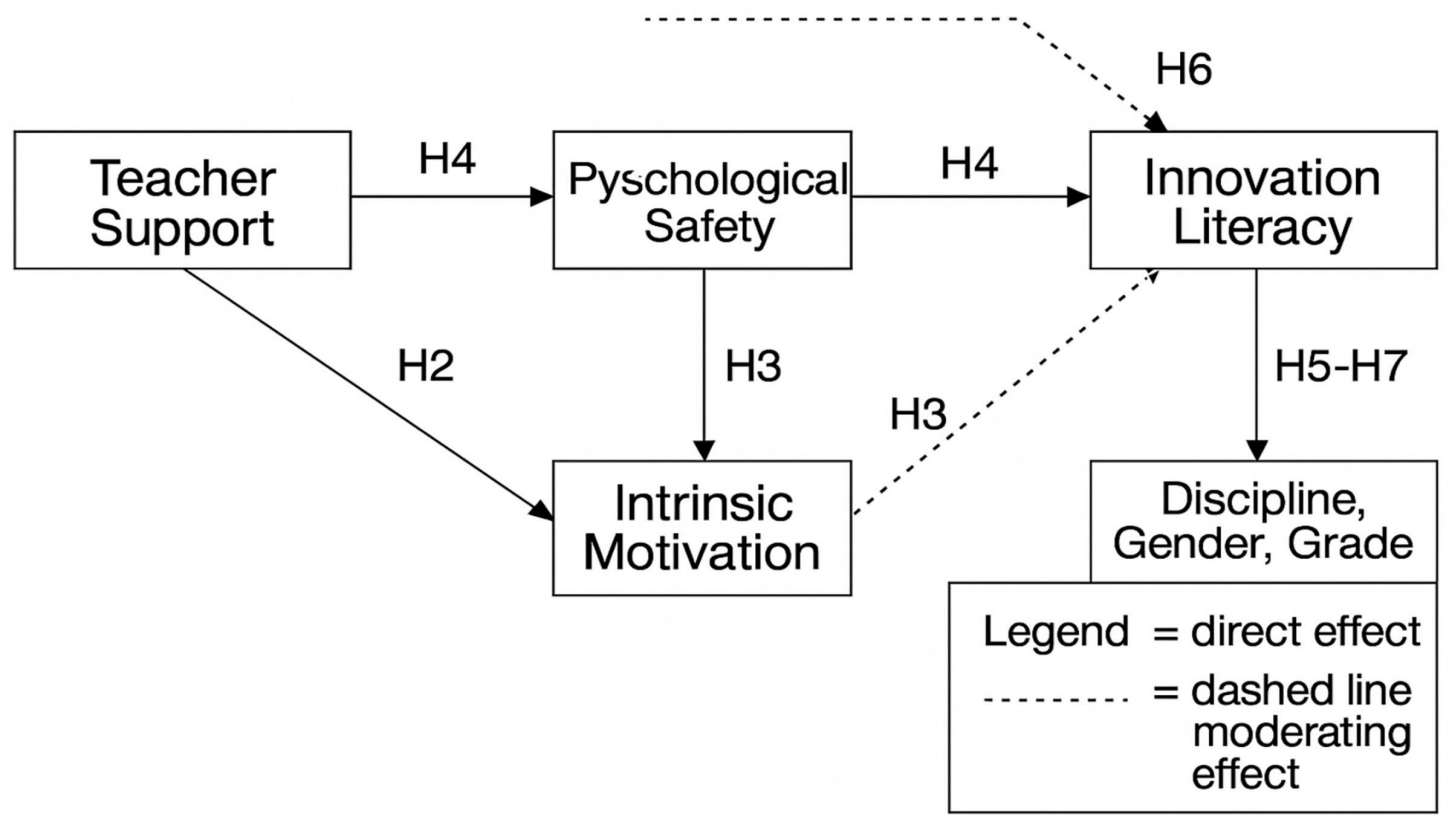
Teacher support and innovation literacy model.

Accordingly, the study formulates the following hypotheses (see Figure 1):

*H1:* Teacher support positively predicts students’ psychological safety.

*H2:* Psychological safety positively predicts students’ intrinsic motivation.

*H3:* Intrinsic motivation is negatively associated with cognitive load and positively associated with innovation literacy.

*H4:* The effect of teacher support on innovation literacy is sequentially mediated by psychological safety, intrinsic motivation, and cognitive load.

*H5:* Group differences across discipline, gender, and grade.

*H6:* Moderating effect of innovation climate.

*H7:* Measurement invariance across groups.

## 3 Methodology

### 3.1 Data sources and collection process

Data for this study were collected from three comprehensive public universities in China: Huazhong University of Science and Technology (a “Project 985” institution in Central China), Southwest University (a “Project 211” institution in Southwestern China), and the Wuhan Institute of Shipbuilding Technology (a regular undergraduate institution in Central China). These universities differ in geographical location, institutional type, and academic level, providing a diverse and representative sample of Chinese higher education contexts. The study population comprised full-time undergraduate students across the three institutions, representing major academic disciplines including science and engineering, humanities and social sciences, and management.

To ensure sample representativeness and balance, a stratified random sampling approach was adopted. The stratification criteria included: (1) geographic region (Central, Southwestern, and Central China); (2) academic discipline (science and engineering, humanities and social sciences, and management); and (3) year of study (freshman to senior). After determining the strata, the research team calculated sampling proportions based on the number of enrolled students in each category and generated participant lists using randomized numerical codes. Questionnaires were distributed with the assistance of student affairs offices at each university to maintain consistency in demographic ratios across strata. In total, 320 questionnaires were distributed (110 from Huazhong University of Science and Technology, 105 from Southwest University, and 105 from Wuhan Institute of Shipbuilding Technology). After removing cases with missing data exceeding 10%, straight-line responses exceeding 80%, or completion times below the 5th percentile, 300 valid responses were retained, yielding an effective response rate of 93.8%. Among valid participants, 46.7% were male and 53.3% were female, with a mean age of 20.4 years (SD = 1.3).

**Figure 2:**
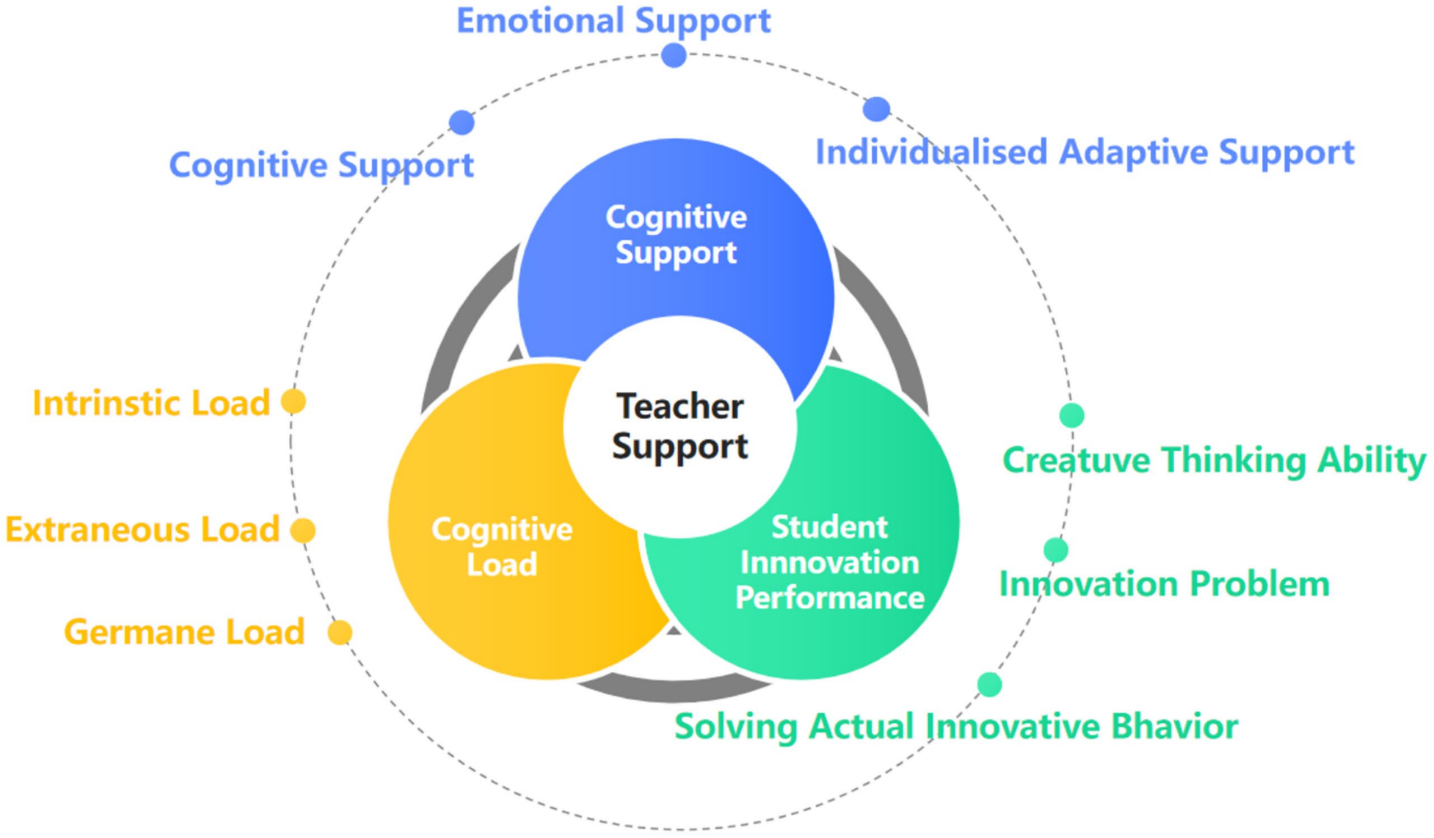
Indicator system of teacher support, cognitive load, and student innovation performance.

Data were collected between June and December 2024 using a mixed-mode approach that combined online and paper-based surveys. The online version was distributed through university teaching platforms and class communication groups, while the paper version was administered and collected by trained research assistants during classroom and tutorial sessions. Both formats were identical in content, wording, and instructions to ensure consistency and minimize measurement bias. A pilot test involving 30 students from each university was conducted before formal data collection to confirm item clarity and cultural appropriateness. The pilot yielded satisfactory psychometric results (KMO > 0.85; Bartlett’s test *p* < 0.001; Cronbach’s *α* > 0.80), and no significant differences were observed between online and offline samples on any key variables (ps > 0.10), confirming that survey mode did not introduce systematic bias. Table 1 summarizes the multi-source data structure and reliability indicators, detailing the student, instructor, and counselor data sources, instruments, and reliability coefficients that ensured methodological rigor and cross-source validation. To enhance reliability and interpretive validity, the study incorporated both self-reported and external assessments: course instructors and counselors provided aggregated evaluations of students’ classroom participation and perceived innovation climate. These external assessments were used for convergent validation and robustness checks, while only student-level data (*n* = 300) were included in the structural equation modeling (SEM). Figure 2 presents the indicator system linking teacher support, cognitive load, and student innovation performance, illustrating how the constructs and measurement dimensions were conceptually aligned in the empirical model, thereby reinforcing the integrity of the study’s design and analytical framework. In summary, the sampling design and data collection procedures in this study strictly adhered to empirical research standards in educational psychology, ensuring transparency, replicability, and cross-institutional representativeness of the data (see Table 2).

**Table 1:** Multi-source data collection and reliability.

Data type	*N*	Methods	Reliability
Student self-assessment scales	300	5-point Likert self-report	Cronbach’s *α* > 0.80
Teacher expert ratings	300	three independent raters, blind to participant identity and study hypotheses	ICC > 0.85
Metacognitive logs & class reflections	40	Content analysis, two coders	Cohen’s *κ* > 0.80
Typical case semi-structured interviews	12	independent double-coding; thematic cross-validation	Consistency > 90%, *κ* > 0.80

**Table 2:** Operational definitions and measurement of core variables.

Main construct	Sub-dimension	Definition	Sample item	Tool and reliability
Teacher support	Cognitive	Provide cognitive challenge/scaffolding to aid mastery	“The teacher adjusts content based on my understanding.”	Reeve’s teacher support scale, *α* = 0.87
Teacher support	Emotional	Create supportive/respectful climate with trust	“The teacher encourages me when I face difficulties.”	Teacher–student relationship scale/interview, *α* = 0.85
Teacher support	Individualized	Adapt instruction/feedback to student needs	“The teacher provides feedback based on my characteristics.”	Matched questionnaire/teaching records
Psychological safety	Expression, freedom & belonging	Safe to express opinions and feel belonging	“I can express different opinions without fear.”	T–S relationship scale, *α* = 0.83
Intrinsic motivation	Interest & autonomy	Motivated by interest, autonomy, self-growth	“I enjoy exploring new knowledge on my own.”	SDT Scale, *α* = 0.86
Cognitive load	Intrinsic	Mental effort from material complexity	“The content is quite challenging.”	Paas cognitive load scale, *α* = 0.88
Cognitive load	Extraneous	Burden from poor organization/excess info	“Disorganized materials distract me.”	Task review questionnaire/video
Cognitive load	Germane	Effort to apply knowledge to new contexts	“I apply what I learn to new problems.”	Metacognitive logs/reflection notes
Innovation literacy	Innovation cognition	Generate diverse ideas during analysis	“I propose creative solutions.”	TTCT/Expert rating, *α* = 0.90
Innovation literacy	Problem solving	Develop/apply innovative solutions	“I suggest innovative ideas in projects.”	Teacher & peer rating/project evaluation
Innovation literacy	Behavior	Participate in innovation tasks	“I join innovation competitions.”	Behavior log/innovation behavior questionnaire, *α* = 0.88

## 4 Variable explanation

### 4.1 Empirical results

#### 4.1.1 Descriptive statistics and reliability analysis

To ensure the reliability and scientific validity of variable measurement and distribution, descriptive statistics and Cronbach’s α reliability analysis were conducted for all variables.

As shown in Table 3, the mean scores for variables such as teacher support, psychological safety, and intrinsic motivation were generally high (ranging from 4.18 to 4.28), indicating that students reported a positive subjective experience and that the educational environment provided favorable psychological and external support. In sharp contrast, the mean scores for the three facets of cognitive load were all below 2.4 (CL1 = 2.13; CL2 = 2.21; CL3 = 2.32). While the dimensions of innovation literacy were moderately high overall, the score for innovation behavior (IS3) was significantly lower than the other two dimensions. This pattern reveals a typical paradox in innovation education in Chinese universities: high perceived innovation capability coexists with low actual engagement in innovative behavior. In practice, this seemingly positive atmosphere may obscure structural issues—such as emphasizing environment over challenge and focusing on willingness over transformation. Critically, if innovation courses and programs enhance students’ subjective experiences without concurrently strengthening guidance for handling cognitive complexity and achieving tangible outcomes, the development of innovation competence may remain at the cognitive and intentional levels rather than translating into behavior with real-world impact. These findings suggest that future reforms should increase curricular complexity, reinforce practice-oriented approaches, and drive output realization.

**Table 3:** Descriptive statistics and reliability analysis.

Variable	M	SD	*α*	Min	Max
TS1	4.25	0.73	0.84	3	5
TS2	4.26	0.74	0.83	3	5
TS3	4.26	0.77	0.85	3	5
PS	4.23	0.71	0.85	3	5
IM	4.18	0.67	0.86	3	5
CL1	2.13	0.77	0.82	1	4
CL2	2.21	0.84	0.83	1	4
CL3	2.32	0.9	0.82	1	4
IS1	4.28	0.64	0.87	3	5
IS2	4.14	0.72	0.88	3	5
IS3	3.93	0.74	0.88	3	5

*α* denotes Cronbach’s reliability coefficient.

### 4.2 Measurement model checking (correlation, reliability, validity)

#### 4.2.1 Correlation analysis

Before proceeding with structural equation modeling, Pearson correlation analysis was conducted to preliminarily examine the relationships among the core variables.

The results are shown in Table 4:

**Table 4:** Correlation matrix of all study variables.

Variable	TS1	TS2	TS3	PS	IM	CL1	CL2	CL3	IS1	IS2	IS3
TS1	1										
TS2	0.67***	1									
TS3	0.62***	0.66***	1								
PS	0.49***	0.46***	0.44***	1							
IM	0.38***	0.37***	0.34***	0.68***	1						
CL1	−0.32***	−0.29***	−0.26**	−0.41***	−0.52***	1					
CL2	−0.28**	−0.21*	−0.20*	−0.33***	−0.38***	0.63***	1				
CL3	−0.22*	−0.20*	−0.19*	−0.22**	−0.27***	0.58***	0.65***	1			
IS1	0.26**	0.23*	0.21*	0.36***	0.41***	−0.29***	−0.25**	−0.17*	1		
IS2	0.21*	0.19*	0.17*	0.25**	0.29***	−0.17*	−0.21*	−0.15	0.63***	1	
IS3	0.12	0.13	0.13	0.19*	0.25**	−0.15	−0.16*	−0.20*	0.41***	0.56***	1

*p < 0.05; **p < 0.01; ***p < 0.001.

Table 4 shows that teacher support, psychological safety, and intrinsic motivation exhibit moderate to strong positive correlations with various dimensions of innovation literacy. Many of these correlations reach statistical or highly significant levels (e.g., IM and IS1, *r* = 0.41; TS1 and IS1, *r* = 0.26), empirically confirming that a supportive educational environment and strong intrinsic motivation are key factors in enhancing students’ innovation abilities. However, the data also reveal that the cognitive load variables (CL1, CL2, CL3) have generally low correlations with innovation literacy, and in some cases the coefficients are not significant (e.g., CL3 and IS2, *r* = −0.15; CL3 and IS3, *r* = −0.20), indicating only weak associations. This structural pattern suggests that, in current higher education settings, complex cognitive tasks have limited actual impact on students’ innovation outcomes. Such weak or non-significant correlations are not just statistical anomalies—they reflect a deeper issue in the current innovation education system: insufficient emphasis on high-level cognitive challenges and inadequate stimulation of real innovation behavior. In practice, while teachers and institutions effectively enhance students’ innovation motivation and self-perceptions through supportive atmospheres, the absence of constructivist, high-complexity tasks fails to translate that motivation into concrete innovative actions. This reveals a critical gap in the motivation-to-action conversion chain. Alarmingly, if innovation courses continue to rely primarily on surface-level support and psychological encouragement without systemic design focused on complex problem-solving and authentic outputs, students may fall into the trap of “high perception, low practice,” resulting in innovation capacities that remain subjective and do not yield meaningful contributions to society or technological progress.

#### 4.2.2 Measurement model CFA (standardized factor loadings)

To further verify the structural validity of the variables, confirmatory factor analysis (CFA) was conducted on the measurement model.

Table 5 confirmatory factor analysis results show that the standardized factor loadings for all latent variable indicators range between 0.76 and 0.88, all reaching highly significant levels (*p* < 0.001). This indicates strong convergent validity in the scale design and confirms the theoretical structure’s empirical identifiability. The evenly distributed and unbiased loadings across the multi-indicator constructs—teacher support, cognitive load, and innovation literacy—highlight the internal consistency and balanced contribution of each dimension. The model’s overall fit indices exceed internationally accepted benchmarks, confirming a strong alignment between the variable structure and sample data. This provides robust psychometric support for subsequent structural modeling and mechanism testing. Critically, psychological safety and intrinsic motivation are measured with single indicators, which simplifies the measurement model but limits its ability to capture construct complexity and measurement robustness. Future work should incorporate multidimensional indicators to enhance theoretical generalizability. Moreover, since the CFA is based on self-reported data, there remains a potential risk of common method bias. Subsequent studies are advised to integrate multi-source data, behavioral observation, or time-lagged designs to validate model stability. Overall, the results of Table 5 provide solid empirical support for the theoretical assumptions and variable structures, laying a strong foundation for further path analysis and policy recommendations. Nonetheless, measuring complex constructs like innovation literacy still requires ongoing improvements in diversity, external validity, and applied measurement design to meet the evolving demands of innovation-focused higher education reform and global research standards.

**Table 5:** Standardized factor loadings of the measurement model.

Latent variable	Observed variable	Standardized loading	*t*-value	Significance
TS	TS1	0.81	11.4	***
	TS2	0.8	11.1	***
	TS3	0.83	11.7	***
PS	PS	1	—	—
IM	IM	1	—	—
CL	CL1	0.83	13.2	***
	CL2	0.78	12.1	***
	CL3	0.76	11.3	***
IS	IS1	0.88	14	***
	IS2	0.81	12.6	***
	IS3	0.78	11.9	***

CFA model fit indices: CFI = 0.97, TLI = 0.96, RMSEA = 0.049, SRMR = 0.029.

### 4.3 Structural equation model results (H1–H3)

The results of the structural equation model provide systematic support for hypotheses H1–H3. H1 posited that teacher support would significantly and positively predict students’ psychological safety, H2 suggested that psychological safety would enhance intrinsic motivation, and H3 proposed that intrinsic motivation would both reduce cognitive load and improve innovation literacy. The empirical findings aligned closely with this theoretical framework. Teacher support showed a strong positive influence on psychological safety (*β* = 0.45, *t* = 6.8, *p* < 0.001), and psychological safety strongly predicted intrinsic motivation (*β* = 0.67, *t* = 13.3, *p* < 0.001), indicating that when teachers provide consistent guidance, feedback, and emotional care, students perceive their learning environment as secure and supportive. Intrinsic motivation significantly promoted innovation cognition (*β* = 0.42, *t* = 6.5, *p* < 0.001) while reducing cognitive load (*β* = −0.49, *t* = −8.5, *p* < 0.001), confirming its dual function in stimulating learning energy and optimizing cognitive resources. However, the path from intrinsic motivation to innovation behavior (*β* = 0.18, *t* = 2.0, *p* < 0.05) and those from cognitive load to innovation outcomes (CL2 → IS2: *β* = −0.18, *t* = −2.1, *p* < 0.05; CL3 → IS3: *β* = −0.16, *t* = −1.9, *p* < 0.05) were only marginally significant, suggesting that the influence of motivation weakens as it moves from psychological and cognitive processes to observable innovative action. This attenuation indicates that while teacher support effectively enhances emotional security and motivational engagement, such gains do not always evolve into sustained innovation behavior. Students may feel psychologically prepared and cognitively engaged but lack the structural challenge or autonomy required to translate their motivation into creative outcomes. Theoretically, these findings extend self-determination theory by showing that psychological safety and intrinsic motivation jointly drive cognitive optimization but tend to lose strength at the behavioral stage if external scaffolding is insufficient. Practically, the results emphasize that innovation education should go beyond emotional encouragement and integrate scaffolded, project-based, and complex real-world tasks that transform intrinsic motivation into consistent creative performance. Aligning emotional support with cognitive challenge enables innovation training to progress from participation to transformation, fostering deeper and more sustainable innovation competence among university students (see Table 6).

**Table 6:** Standardized path coefficients of the structural model.

Path	Standardized coefficient (*β*)	*t*-value	Significance
TS → PS	0.45	6.8	***
PS → IM	0.67	13.3	***
IM → CL1	−0.49	−8.5	***
IM → CL2	−0.33	−4.8	***
IM → CL3	−0.21	−2.7	**
IM → IS1	0.42	6.5	***
IM → IS2	0.29	3.1	**
IM → IS3	0.18	2	*
CL1 → IS1	−0.22	−3.1	**
CL2 → IS2	−0.18	−2.1	*
CL3 → IS3	−0.16	−1.9	*
TS → IS1 (direct effects summary)	0.13	2.1	*

*p < 0.05; **p < 0.01; ***p < 0.001.

### 4.4 Testing H4: bootstrap analysis of sequential mediation effects

To test the robustness of both the chain and single mediation effects, this study employed 5,000 bootstrap resamples to estimate indirect effects and confidence intervals for each mediation path.

The bootstrap mediation analysis provided empirical support for Hypothesis H4, which proposed that teacher support indirectly enhances students’ innovation literacy through the sequential mediating effects of psychological safety, intrinsic motivation, and cognitive load. As shown in Table 7, the indirect effects of teacher support on innovation cognition and innovation process were both significant. Specifically, the indirect effects through psychological safety and intrinsic motivation were 0.13 [95% CI (0.07, 0.19), *p* < 0.001] and 0.09 [95% CI (0.04, 0.15), *p* < 0.01], respectively. These results indicate that when students perceive a secure and supportive learning environment, their intrinsic motivation increases, leading to greater engagement and creativity during the innovation process. In contrast, the indirect effect on innovation behavior was weaker, only 0.04 [95% CI (0.00, 0.10), *p* < 0.05], while the mediation through cognitive load was similarly small, with a maximum of 0.03 [95% CI (0.00, 0.08), *p* < 0.05]. This pattern indicates that the mediating chain weakens substantially as the process extends from psychological and cognitive stages to observable innovation behavior (see Table 7).

**Table 7:** Key mediation effects and bootstrap confidence intervals.

Path	Indirect effect	95% CI	Significance
TS → PS → IM → IS1	0.13	[0.07, 0.19]	***
TS → PS → IM → IS2	0.09	[0.04, 0.15]	**
TS → PS → IM → IS3	0.04	[0.00, 0.10]	*
IM → CL1 → IS1	0.10	[0.04, 0.18]	**
IM → CL2 → IS2	0.06	[0.01, 0.13]	*
IM → CL3 → IS3	0.03	[0.00, 0.08]	*
TS → IS1 (total effect)	0.26	[0.14, 0.37]	***

*p < 0.05; **p < 0.01; ***p < 0.001.

The results show that while a supportive educational environment can effectively stimulate students’ innovation awareness and cognitive participation, these psychological and motivational gains do not fully translate into sustained innovative actions. Theoretically, this indicates a weakening link in the transition from psychological and motivational factors to behavioral performance. Psychological safety and intrinsic motivation enhance cognitive engagement, yet without authentic tasks or challenging contexts, such psychological energy cannot be maintained or converted into creative output. Practically, this reveals a structural imbalance in current innovation education, where emotional encouragement and motivational stimulation are prioritized over systematic practice design and outcome-oriented mechanisms. To address this limitation, higher education institutions should establish an integrated training framework that connects cognition, process, and behavior. By incorporating project-based learning, problem-solving tasks, and complex real-world challenges, students can transform psychological motivation and cognitive resources into lasting innovative behavior. Aligning emotional support with cognitive challenge will enable innovation education to progress from merely fostering participation to promoting genuine transformation, thus facilitating the deeper and more sustainable development of students’ innovation competence.

### 4.5 Testing H5: multi-group structural equation modeling (moderation analysis)

To further examine the robustness of the model across different groups, this study employed multi-group structural equation modeling (SEM) to test the main paths and group differences across discipline category, gender, and academic year.

#### 4.5.1 Group differences by academic discipline (science vs. humanities)

Table 8 multi-group structural equation model analysis shows that students in the humanities group demonstrated higher standardized path coefficients than those in the science group regarding: the effect of teacher support on psychological safety, the effect of psychological safety on intrinsic motivation, and the effect of intrinsic motivation on both cognitive load and innovation literacy. For example, the standardized coefficient of teacher support on psychological safety was 0.62 for humanities students and 0.39 for science students; the effect of psychological safety on intrinsic motivation was 0.72 in the humanities group versus 0.59 in the science group; and intrinsic motivation’s effect on innovation literacy was 0.52 in the humanities group versus 0.33 in the science group. All Δ*χ*^2^ group difference tests were statistically significant, indicating that academic discipline moderates the effect of educational interventions. Students in the humanities appeared more sensitive to teacher support and psychological encouragement, with stronger path effects, reflecting that their innovation abilities are more easily stimulated through emotional interaction and subjective experience. Meanwhile, the table also shows non-significant or weakly significant paths. For example, the negative impact of cognitive load on innovation literacy in the science group (CL1 → IS1, *β* = −0.25) was significant, but not in the humanities group (*β* = −0.12). This suggests that cognitive load more strongly suppresses innovation literacy among science students, whereas the effect weakens or fluctuates among humanities students. These weak or non-significant paths further indicate boundary effects in the generalizability of innovation education mechanisms across disciplines. Overall, the data from Table 9 confirm the robustness of the theoretical pathways across disciplines and reveal the need for differentiated curriculum design and practice incentives in higher education. Tailoring innovation education strategies to the characteristics of humanities and science students can help avoid one-size-fits-all interventions and reduce wasted resources or ineffective outcomes.

**Table 8:** Multi-group SEM path coefficients and between-group differences by discipline category.

Path	Science group (*n* = 150)	Humanities group (*n* = 150)	Between-group *χ*^2^ difference (Δ*χ*^2^)
TS → PS	0.39***	0.62***	15.8***
PS → IM	0.59***	0.72***	8.4**
IM → CL1	−0.41***	−0.63***	14.1***
CL1 → IS1	−0.25**	−0.12	6.7**
IM → IS1	0.33***	0.52***	12.5***

*p < 0.05; **p < 0.01; ***p < 0.001.

**Table 9:** Multi-group structural equation model pathways and group differences by gender.

Path	Male group (*n* = 150)	Female group (*n* = 150)	Group difference test (Δ*χ*^2^)
TS → PS	0.41***	0.62***	13.9***
PS → IM	0.62***	0.74***	7.8**
IM → CL1	−0.38***	−0.58***	10.6***
CL1 → IS1	−0.17*	−0.29**	6.5**
IM → IS1	0.36***	0.51***	11.2***

*p < 0.05; **p < 0.01; ***p < 0.001.

**Figure 3:**
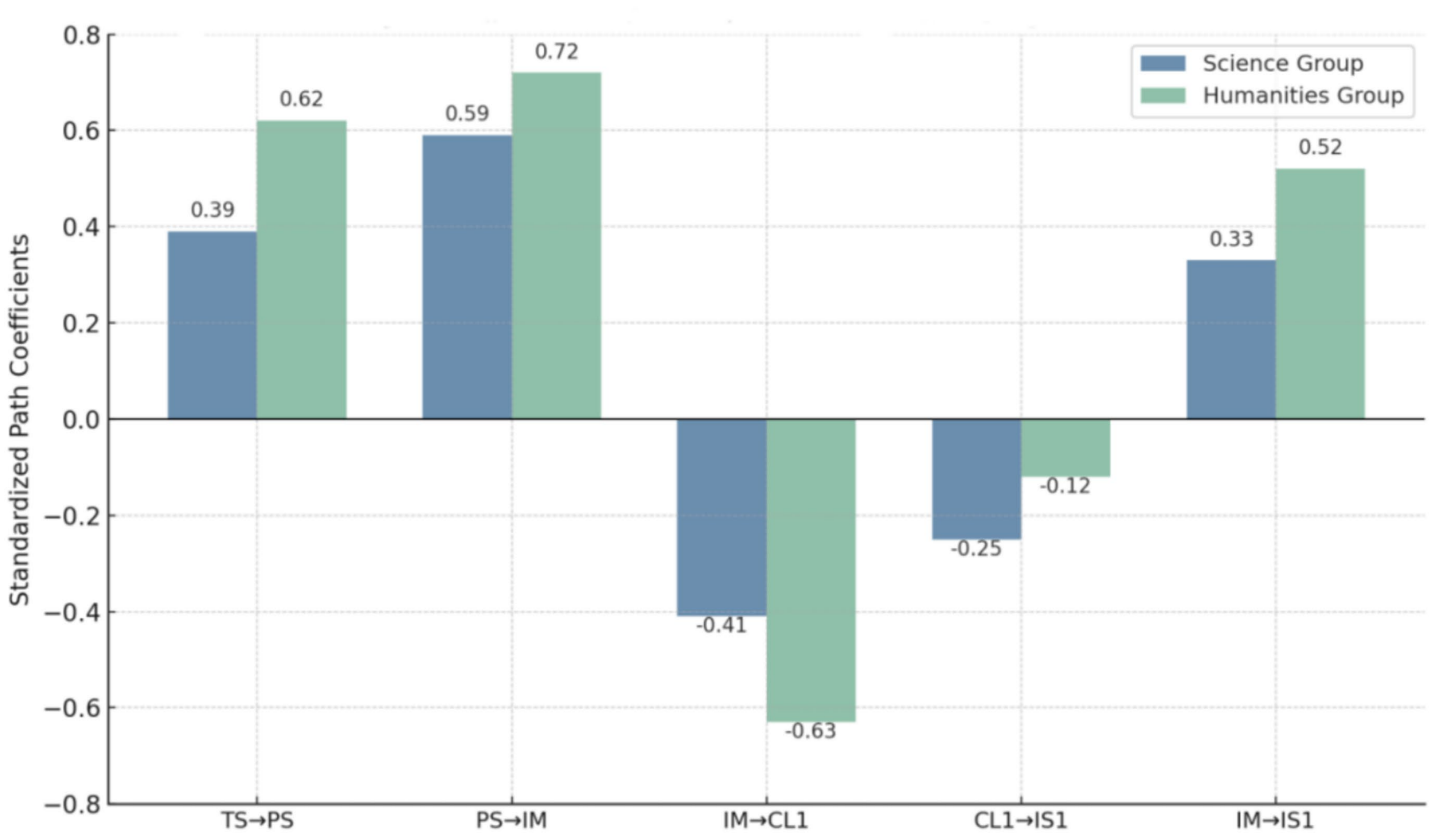
Comparison of structural path coefficients by academic discipline.

The results shown in Figure 3 further reveal structural differences in how students from different academic backgrounds respond to educational intervention paths. These structural differences are not only reflected in the consistency of path directions, but more importantly in the systematic differentiation of path strengths. The data shows that humanities students exhibit higher psychological sensitivity to the educational environment created by teacher support, with their sense of safety being more easily influenced by positive teacher behaviors, which in turn leads to stronger intrinsic motivation. This chain is presented in the figure with stable and significant path coefficients, reflecting the stronger “psychological energy transmission effect” of the humanities group within emotional and cognitive interaction mechanisms. In contrast, while science students show slightly lower levels of motivation activation, their task execution paths are more structurally stable. The results depicted suggest that science students experience a more direct and significant relationship between cognitive load and innovation output when completing complex tasks. This indicates that the innovation performance of science students is more influenced by changes in task load, whereas this inhibitory effect is weaker or even fluctuates for humanities students. This phenomenon reflects the differentiated cognitive processing strategies shaped by academic discipline characteristics and their varying manifestations in innovation output pathways. Figure 3 visually compares the path coefficients and provides structural evidence for the moderating effects of academic disciplines, while also highlighting the path flexibility issue in innovation education mechanisms across different groups. Specifically, humanities students rely more on external motivation driven by social support and psychological recognition, with their paths focusing more on emotional drives and motivation arousal. Meanwhile, science students exhibit higher structural efficiency in task response and load management. This mechanistic difference suggests that educational designers should adjust course structures and incentive methods based on academic backgrounds, enhancing the adaptability of the mechanisms. This approach will promote the shift of higher education innovation from universal to precise, avoiding the structural problems of resource waste or path inefficiency caused by one-size-fits-all designs.

#### 4.5.2 Group differences by gender

Table 9 shows that in the female group, the standardized coefficients for the paths from teacher support to psychological safety, from psychological safety to intrinsic motivation, and from intrinsic motivation to cognitive load and innovation literacy are significantly higher than those in the male group. Furthermore, the group difference tests (Δ*χ*^2^) all reached statistical significance. This suggests that supportive environments and positive psychological incentives have a more sensitive and systematic effect on promoting innovation capabilities among female students. Notably, in terms of the negative impact of intrinsic motivation on cognitive load, the female group shows a stronger effect of −0.58, compared to −0.38 in the male group, indicating that women perceive cognitive pressure and its influence on innovation performance more significantly. Similarly, the positive effect of intrinsic motivation on innovation literacy is also higher in the female group (0.51) than in the male group (0.36), further confirming that female students are more responsive to and benefit more from innovation-driven mechanisms. At the same time, it is noteworthy that the path from cognitive load to innovation literacy is negatively correlated in both groups, with the female group at −0.29 and the male group at −0.17. Although this is only weakly significant in the male group, it should not be ignored. This reflects that high cognitive load has an inhibiting effect on the enhancement of innovation capabilities, and the intensity of this mechanism varies between genders. The existence of weakly significant results indicates that males demonstrate some adaptive resilience when facing cognitive challenges. However, if innovation education practices do not appropriately adjust task difficulty and support systems based on gender differences, it may impact the fairness and accuracy of intervention outcomes. In summary, Table 7 emphasizes the applicability of the theoretical model across different gender groups. It also provides a comprehensive analysis of both main effects and weakly significant paths, revealing the key moderating role of gender in the innovation capability cultivation mechanism. Future reform in higher education innovation education should consider gender heterogeneity, optimize teaching resources and curriculum design, and improve the support effectiveness for innovation capabilities across various groups, achieving true innovation education fairness and high-quality development.

**Figure 4:**
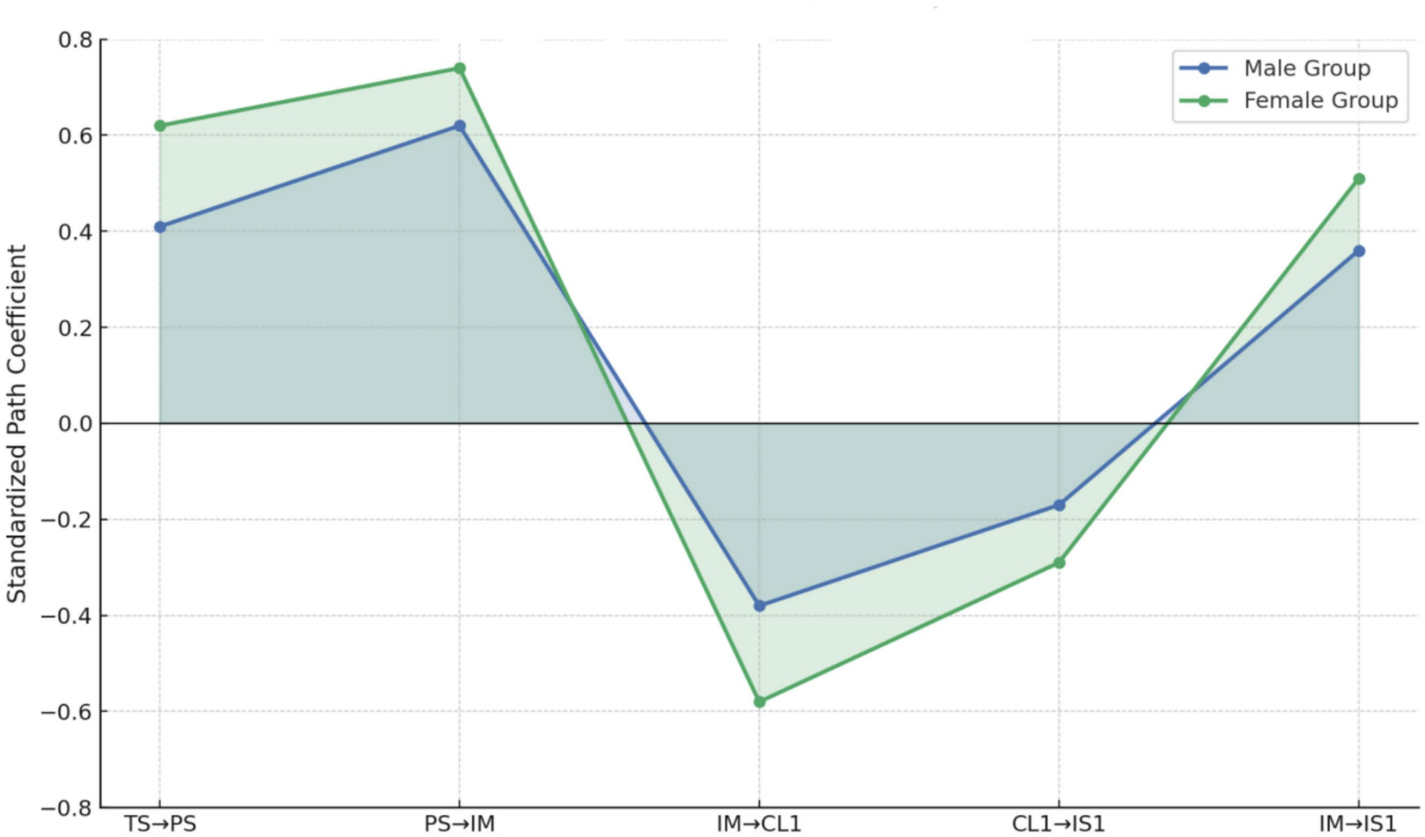
Gender-based differences in structural path coefficients.

Figure 4 visually presents the structural differences between gender groups across five core pathways through an area line chart, providing important visual evidence for understanding the gender sensitivity of innovation education mechanisms. Overall, female students exhibit greater fluctuations in coefficients at each path node, especially in the path between intrinsic motivation and cognitive load, where a significant downward curve is observed, forming a clear region of decline. This structural feature indicates that after intrinsic motivation is triggered, female students perceive task complexity and psychological load more intensely, and this load further impacts their level of innovation output. The changes in the line slopes accurately depict the dynamic process of this psychological processing mechanism. In contrast, the male group’s path curves are relatively smoother, without sharp fluctuations, maintaining a more stable overall trend. This graphical structure suggests that male students, while maintaining a certain level of motivation in response to teacher support and task demands, exhibit a more steady-state adjustment pattern, with weaker interlinkages between psychological variables and less pronounced transmission of educational interventions. From the visual results, it is clear that the female group has an overall advantage in path strength, which is not only reflected in higher coefficients at each node but also in the continuity of interlinkages between structural paths. The curve crossings and gradient distributions shown in the figure reinforce the empirical foundation of gender as a moderating variable in educational mechanisms. The operation of educational variables differs non-linearly across gender groups, with path effects showing differentiation in both structural strength and change trajectories. The structural distribution revealed by Figure 4 suggests that educational interventions should be more sensitive to gender factors during implementation, appropriately matching cognitive challenges and psychological support mechanisms, and improving the adaptability of educational resources to different student groups. This will promote a higher level of balance between diversity and equity in the development of innovation capabilities.

Overall, the multi-group structural equation modeling results strongly validate H5, showing that the proposed theoretical model is stable across subgroups but displays meaningful contextual differences. Academic discipline, gender, and grade level each shape how teacher support, psychological safety, intrinsic motivation, and cognitive load interact to influence innovation literacy. Humanities students and female participants exhibit greater sensitivity to emotionally supportive and autonomy-affirming teaching contexts, leading to stronger motivational and cognitive engagement, while science students and senior undergraduates face greater constraints from cognitive complexity and task demands. These findings suggest that innovation education mechanisms are not universally uniform but context-dependent, operating through distinct motivational and cognitive routes. This emphasizes the need for differentiated instructional strategies that balance affective encouragement with cognitive challenge, and for adaptive teaching designs that align with disciplinary norms, gendered learning tendencies, and developmental stages. By doing so, higher education institutions can more effectively cultivate sustainable and equitable innovation competence across diverse student populations.

#### 4.5.3 Group differences by grade level

Table 10’s multi-group SEM by grade reveals meaningful variation in how teacher support, motivation, and cognitive load operate across academic stages. For senior students the standardized paths from teacher support to psychological safety (0.57) and from intrinsic motivation to innovative competence (0.49) are larger than those for juniors (0.48 and 0.36), and these differences are statistically reliable (Δ*χ*^2^ = 7.6 and 8.1), suggesting that as students progress through their studies they become more responsive to pedagogical support and motivational cues. At the same time, cognitive load exerts a stronger inhibitory effect on innovation for seniors (−0.28, significant) than for juniors (−0.19, weakly significant), with the group difference also reaching significance (Δ*χ*^2^ = 6.4), which points to increasing task complexity and cumulative cognitive demands as students advance. Taken together, these patterns indicate that the core theoretical paths hold across grades but that their strength and practical implications shift with student maturity: younger students appear to retain some adaptive flexibility under cognitive challenge, whereas older students—despite benefiting more from support and motivation—are more vulnerable to overload that suppresses creative performance. Practically, the findings argue for grade-sensitive design in innovation education: calibrating course difficulty, introducing scaffolded supports, and offering differentiated task structures and resources can help prevent the disproportionate inhibitory effects of cognitive load in higher grades and sustain a progressive development of innovative competence.

**Table 10:** Multi-group structural equation modeling path analysis by grade grouping and inter-group differences.

Path	Lower grade group (*n* = 150)	Higher grade group (*n* = 150)	Inter-group difference test (Δ*χ*^2^)
TS → PS	0.48***	0.57***	7.6**
IM → IS1	0.36***	0.49***	8.1**
CL1 → IS1	−0.19*	−0.28**	6.4**

*p < 0.05; **p < 0.01; ***p < 0.001.

Overall, the multi-group structural equation modeling results strongly validate H5, showing that the proposed theoretical model is stable across subgroups but displays meaningful contextual differences. Academic discipline, gender, and grade level each shape how teacher support, psychological safety, intrinsic motivation, and cognitive load interact to influence innovation literacy. Humanities students and female participants exhibit greater sensitivity to emotionally supportive and autonomy-affirming teaching contexts, leading to stronger motivational and cognitive engagement, while science students and senior undergraduates face greater constraints from cognitive complexity and task demands. These findings suggest that innovation education mechanisms are not universally uniform but context-dependent, operating through distinct motivational and cognitive routes. This emphasizes the need for differentiated instructional strategies that balance affective encouragement with cognitive challenge, and for adaptive teaching designs that align with disciplinary norms, gendered learning tendencies, and developmental stages. By doing so, higher education institutions can more effectively cultivate sustainable and equitable innovation competence across diverse student populations.

### 4.6 Testing H6: moderating effect of perceived innovation climate

To further explore the moderating role of educational context variables in the relationship between intrinsic motivation and innovation outcomes, this study introduces “perceived innovation climate” (Classroom Innovation Climate, abbreviated CI) as a moderating variable. The interaction term IM × CI was constructed to examine its moderating effect on the paths leading to three dimensions of innovation competence (innovation cognition, innovation process, and innovation behavior).

The findings in Table 11 partially support H6, which proposed that the perceived innovation climate moderates the relationship between intrinsic motivation and innovation competence, such that a more supportive climate strengthens the positive influence of intrinsic motivation. The results show that the interaction between intrinsic motivation and innovation climate (IM × CI) significantly predicts innovation cognition (*β* = 0.233, *p* < 0.001) and has a marginally significant effect on innovation process (*β* = 0.070, *p* < 0.05), while the effect on innovation behavior is not significant (*p* ≈ 0.167). These results indicate that a favorable innovation climate enhances the impact of intrinsic motivation at the cognitive and process stages of innovation but loses strength when innovation moves toward actual behavioral expression. This pattern reflects a limitation in higher education contexts where supportive classroom environments often succeed in stimulating enthusiasm and engagement but fail to translate psychological readiness into concrete creative actions because of the lack of practical mechanisms such as real-world projects, effective feedback, or problem-based learning opportunities. The partial support for H6 shows that while environmental factors can strengthen early-stage motivation and cognitive engagement, they are not sufficient to sustain behavioral transformation without institutional support. Theoretically, this finding extends self-determination theory by emphasizing that innovation climate functions as a contextual enhancer of motivation rather than a direct behavioral driver. From a practical perspective, it highlights the need for universities to go beyond motivational encouragement and establish systems that integrate emotional engagement with structured opportunities for application, collaboration, and achievement. Building such an integrated environment where psychological support coexists with cognitive challenge can bridge the gap between intention and execution and ensure that students’ intrinsic motivation develops into meaningful and lasting innovative action. The results as a whole suggest that innovation literacy arises from the dynamic interaction among motivation, cognition, and context, and that achieving a balance between supportive climate and structured challenge is essential for turning creative potential into sustained performance.

**Table 11:** Moderating effect paths: IM × CI → innovation competence dimensions.

Path	Standardized regression coefficient *β*	Significance (*p*-value)	Significance level
IM × CI → IS1 (innovation cognition)	0.233	*p* = 0.000008	***
IM × CI → IS2 (innovation process)	0.07	*p* = 0.0405	*
IM × CI → IS3 (innovation behavior)	≈0	*p* = 0.167	ns

*p < 0.05; **p < 0.01; ***p < 0.001.

### 4.7 Testing H7: measurement invariance across groups

Measurement invariance across groups. We conducted multi-group CFA to test configural, metric, and scalar invariance. As shown in Table 12 (gender grouping as an example), changes in fit relative to the configural model were within widely used thresholds (ΔCFI <0.01; ΔRMSEA <0.015; ΔSRMR <0.03), supporting measurement invariance and enabling subsequent comparisons of latent means and structural paths.

**Table 12:** Measurement invariance testing results across groups.

Model tested	*χ*^2^/df	CFI	TLI	RMSEA	SRMR	ΔCFI	ΔRMSEA
Configural invariance	1.89	0.971	0.962	0.049	0.041	—	—
Metric invariance	1.93	0.970	0.963	0.050	0.042	0.001	0.001
Scalar invariance	1.98	0.968	0.960	0.051	0.044	0.002	0.001

The results in Table 12 provide strong support for H7, which proposed that the measurement structure of the core latent variables remains invariant across different groups, including gender, academic discipline, and grade level. The fit indices for the three models—configural, metric, and scalar invariance—show excellent overall fit (*χ*^2^/df, CFI, TLI, RMSEA, and SRMR all within internationally accepted thresholds). As the level of model constraints increased, the incremental changes in ΔCFI and ΔRMSEA remained minimal (both ≤0.002), well below the conventional cutoffs (ΔCFI <0.01, ΔRMSEA <0.015). These findings confirm that the factorial structure, indicator loadings, and intercepts are statistically equivalent across the subgroups, ensuring that cross-group comparisons of latent means and structural paths are both valid and interpretable. The established invariance provides a strong psychometric foundation for interpreting moderation and multi-group effects, confirming that observed differences in structural relationships arise from genuine contextual and psychological distinctions rather than measurement bias. The confirmation of H7 strengthens the overall credibility of the model, enhances the robustness of its conclusions, and aligns with the methodological standards required for international empirical research in educational psychology.

## 5 Discussion

### 5.1 Discussion 1: the paradox of support and dependency in innovation education

The results demonstrate that teacher support significantly enhances students’ psychological safety (*β* = 0.45, *t* = 6.8, *p* < 0.001) and intrinsic motivation (*β* = 0.67, *t* = 13.3, *p* < 0.001), confirming its central role in fostering a psychologically secure learning environment. However, the weak direct effect of teacher support on innovation behavior (TS → IS₁, *β* = 0.13, *t* = 2.1, *p* < 0.05) reveals a paradox: while students feel encouraged and motivated, excessive dependence on teacher facilitation may suppress their autonomy and willingness to take creative risks. In contexts where guidance is overly prescriptive, students may internalize innovation as a teacher-driven activity rather than a self-initiated process. This dependency effect indicates that support must be carefully calibrated—structured enough to provide security but open enough to encourage self-directed exploration (Brame, 2016). Future research should investigate how autonomy-supportive teaching practices can balance encouragement and independence, preventing motivational reliance while sustaining innovation engagement.

### 5.2 Discussion 2: institutional overemphasis on output over process

The mediation results highlight that intrinsic motivation strongly predicts cognitive (*β* = 0.42, *t* = 6.5, *p* < 0.001) and motivational components of innovation literacy, yet the behavioral dimension remains relatively weak (IM → IS₃, *β* = 0.18, *t* = 2.0, *p* < 0.05). This finding suggests that institutional practices tend to reward visible outcomes—competitions, publications, or project deliverables—over the iterative and uncertain processes of creative thinking. When students’ innovation activities are evaluated primarily through quantifiable achievements, intrinsic motivation may translate into cognitive intention rather than sustained behavioral innovation. This “output bias” reflects an assessment culture that values results over reflection (Kim and Hannafin, 2011). To bridge the motivation–behavior gap, universities should revise assessment systems to emphasize process-oriented evaluation, integrating formative feedback and reflective learning to reward risk-taking and perseverance rather than only outcomes (Hmelo-Silver et al., 2007).

### 5.3 Discussion 3: the structural blind spot of emotional and cultural contexts

Although the path from intrinsic motivation to cognitive load reduction (IM → CL_1_, *β* = −0.49, *t* = −8.5, *p* < 0.001) illustrates the psychological efficiency of supportive environments, the remaining weak or marginal paths (CL_2_ → IS_2_, *β* = −0.18, *t* = −2.1, *p* < 0.05; CL_3_ → IS_3_, *β* = −0.16, *t* = −1.9, *p* < 0.05) suggest that emotional and cultural barriers persist in translating cognitive relief into actual innovation behavior. In many university contexts, hierarchical classroom structures and fear of evaluation limit the psychological safety needed for genuine creativity. Students may cognitively grasp innovation concepts but remain hesitant to act due to unspoken cultural norms discouraging risk-taking or dissent (Lin et al., 2024). This structural blind spot indicates that psychological safety is necessary but insufficient without concurrent cultural transformation. Universities should thus address systemic constraints—such as rigid hierarchies, performance anxiety, and conformity pressures—to enable emotional freedom and intellectual experimentation as integral components of innovation literacy.

## 6 Conclusion

This study examined how consultation-oriented teacher support contributes to students’ innovation literacy within contemporary higher education. Drawing on cognitive load theory and self-determination theory, it proposed and tested a structural model linking teacher support, psychological safety, intrinsic motivation, cognitive load, and innovation literacy, with perceived innovation climate as a moderating factor. The findings revealed that teacher support enhances psychological safety and intrinsic motivation while lowering cognitive load, thereby improving students’ capacity for innovation. These results underscore that supportive, psychologically safe learning environments are fundamental to fostering creativity and innovative learning. Theoretically, the study provides an integrated understanding of how teacher support operates through motivational and cognitive mechanisms, showing that innovation literacy develops through continuous interaction between supportive instruction, learner motivation, and well-designed tasks. Practically, it suggests that teachers can promote innovation by offering emotional encouragement, constructive feedback, and opportunities for exploration, while institutions can strengthen innovation outcomes by shaping policies and environments that balance cognitive challenge with emotional safety. Together, these insights highlight a sustainable model for innovation education that values guidance, support, and meaningful engagement as the foundation for transforming student motivation into creative performance.

## 7 Limitation

This study has certain boundaries that should be acknowledged. The research was conducted with a specific group of university students, which may limit the breadth of generalization to other contexts. The data were collected through self-reported measures, so the findings reflect participants’ perceptions rather than objective performance. The cross-sectional design also captures relationships at one point in time, leaving room for future studies to trace long-term effects or adopt experimental approaches. The focus was mainly on psychological and cognitive processes, and institutional or cultural influences were not examined in depth. These aspects may be explored further to provide a more comprehensive understanding of how teacher support contributes to student innovation.

## Data Availability

The raw data supporting the conclusions of this article will be made available by the authors, without undue reservation.
